# Assessing Natural Fillers as Substitutes for Glass Fibers in Polyamide 6 Composites for Large-Format Additive Manufacturing

**DOI:** 10.3390/polym18091049

**Published:** 2026-04-26

**Authors:** Alessandro Sorze, Francesco Valentini, Sofia Santi, Matteo Perini, Nicole Soligo, Mauro Buccella, Laura Pasquardini, Andrea Dorigato

**Affiliations:** 1 Department of Industrial Engineering and INSTM Research Unit, University of Trento, Via Sommarive 9, 38123 Trento, Italy; 2 ProM Facility of Trentino Sviluppo S.p.A., Via F. Zeni 8, 38068 Rovereto, Italy; 3 Tessilquattro S.p.A., Via Linfano 9, 38062 Arco, Italy; 4 Indivenire Srl, Via Sommarive 18, 38123 Trento, Italy; 5 Department of Engineering, University of Campania Luigi Vanvitelli, Via Roma 29, 81031 Aversa, Italy

**Keywords:** polyamide 6, natural fillers, glass fibers, composites, additive manufacturing, printability

## Abstract

This work investigated the potential of different natural fillers, i.e., clay, calcium carbonate, and silica, as sustainable alternatives to glass fibers (GFs) in polyamide 6 (PA6) for Large-Format Additive Manufacturing (LFAM) applications in order to guarantee the chemical recyclability of the produced materials. Specifically, PA6-based composites containing ≤ 10 wt% natural fillers were compared with a conventional system (30 wt% GF-reinforced PA6) from rheological, morphological and thermo-mechanical perspectives. Rheological analysis showed that silica- and clay-filled samples displayed similar rheological response to the GF-filled reference due to their large particle size. Thermal analyses revealed a slight increase in crystallinity (up to 32%) for filled samples, indicating a potential nucleating effect of the natural fillers. Calcium carbonate-filled composites achieved thermal conductivity values comparable to the GF-filled reference (≥0.42 W/mK) indicating a high heat dissipation capability during printing operations. Morphological analysis performed on preliminary LFAM components revealed satisfactory printing quality and good filler dispersion. Flexural tests showed that silica and calcium carbonate could provide a balanced mechanical response, thereby reducing the anisotropy of printed components. These results demonstrated that the addition of suitable natural fillers at limited concentrations (≤10 wt%) can represent a lightweight and eco-sustainable alternative to GF reinforcement in LFAM applications.

## 1. Introduction

In recent years, the market for Additive Manufacturing (AM) has grown considerably as it allows components to be built up layer by layer, with material only deposited where required [1,2]. Concurrently, numerous industries in the field of polymeric materials have recognized the need for Large-Format Additive Manufacturing (LFAM), an extrusion-based technique used to fabricate large-scale, structurally robust components for aerospace, automotive, energy and buildings applications, in order to facilitate cost-effective mass customization [3,4]. Furthermore, its capacity to rapidly produce geometrically complex shapes within large printed volumes provides a strategic advantage over conventional subtractive and forming processes, especially when short production times or functional prototypes are required [4,5,6]. Despite these benefits, LFAM presents several processing challenges. The absence of a heating chamber and the high temperature gradients within the printed structure can often lead to anisotropic shrinkage, which in turn causes warping, delamination, cross-sectional tapering, sagging or structural distortion [7,8,9]. The issue is aggravated in semicrystalline polymers, where rapid cooling amplifies differential shrinkage between layers [4]. A possible strategy to minimize shrinkage involves incorporating fillers or reinforcements, either in fiber or particulate form, in the polymer matrix, acting as physical barriers to restrict macromolecular mobility [10]. Previous studies have shown that materials with higher thermal stability, such as carbon fiber (CF)-reinforced acrylonitrile butadiene styrene (ABS), followed by glass fiber (GF)-reinforced ABS, retain dimensional stability at elevated temperatures more effectively than unfilled polymers [11]. Rheological investigations of high-performance polymers, including polyphenylsulfone (PPSU), polyethersulfone (PES), and polyphenylene sulfide (PPS) further demonstrated that the addition of reinforcing fibers significantly modifies melt viscosity and extrusion torque requirements [12]. Ajinjeru et al. [13] demonstrated that the addition of CF to polyetherimide (PEI) resulted in an enhanced shear-thinning effect and a substantial increase in viscosity. Even if fiber reinforcements can often effectively counteract thermal expansion and shrinkage, their orientation remains largely in-plane during deposition, meaning that fibers rarely bridge layers and thus do not directly improve interlayer adhesion.

Moreover, some authors demonstrated that replacing the most common filament materials using pellet-fed extrusion systems [14,15,16,17] offers higher deposition rates (often by two orders of magnitude), reduced material cost, and lower energy consumption per kilogram of printed materials, though at the price of increased system complexity. Among engineering polymers suitable for LFAM, polyamide 6 (PA6) is widely used thanks to its excellent mechanical strength, thermal resistance, and processability [18,19,20,21,22]. However, PA6 is highly hygroscopic, and its mechanical properties are strongly affected by moisture uptake, making reinforcement essential for stable performance in demanding environments [23]. Glass fibers are conventionally considered to be among the best options for improving stiffness and dimensional stability when incorporated in PA6 matrix [24,25,26]. Indeed, GF-reinforced thermoplastic pellets for 3D printing are widely adopted in pellet-fed AM systems, yielding much higher strength than unfilled plastics [27,28]. However, the extensive use of GF presents several drawbacks, including increased composite density, high processing energy, elevated nozzle wear, and limited recyclability, raising concerns regarding the sustainability of the components at their end of life.

Consequently, in recent years, research efforts have focused on integrating natural fillers as potential GF substitutes in PA6-based composites [29,30]. Such fillers, including layered silicates, carbonates, and siliceous minerals, offer advantages such as lower cost, natural abundance, reduced environmental footprint and, in some cases, intrinsic functionalities such as improved thermal stability or barrier properties [31,32]. Furthermore, it has been demonstrated that natural fillers, such as organically modified montmorillonite, can easily be exfoliated into the PA6 matrix, thereby providing efficient thermo-mechanical properties [33]. Despite these advantages, their reinforcing efficiency compared to glass fibers remains under-explored, particularly when used as fillers in PA6 for LFAM applications, where filler–matrix interaction strongly influences both processability and final performance. While the effects of the above-mentioned fillers have been separately studied in various polymers, comparisons under consistent processing conditions, particularly within a PA6 matrix and in a LFAM-relevant framework, remain scarce. Therefore, this paper addresses this knowledge gap by systematically investigating the potential of different natural fillers, including clay, talc, calcium carbonate, kaolin, silica and wollastonite, as alternatives to glass fibers in PA6 matrices for LFAM applications. These natural fillers were chosen according to their availability on the market and suitability for industrial LFAM production. In order to guarantee the chemical recyclability of the resulting materials at their end of life, a limited filler loading (i.e., lower than 10 wt%) has been considered. Indeed, a potential end-of-life route for PA6-based composites is low-temperature acid depolymerization. This process consists of two main stages: depolymerization and purification. During the first stage, the polymer chains are cleaved to form cyclic monomer caprolactam with minor amounts of oligomeric by-products. The second stage involves purification of the recovered caprolactam, typically though distillation, to remove residual impurities. This purification step is facilitated in the presence of natural fillers compared to glass fibers, as these fillers tend to sediment more readily, simplifying their separation from the depolymerized product. This study combines rheological analysis with thermo-mechanical characterization to provide new insights into the potential of natural fillers as sustainable reinforcing agents for PA6 components manufactured through LFAM. A systematic comparison with the compound traditionally used for this application (i.e., PA6 with 30 wt% GF) has been carried out. The most promising formulations were then used to print prototypes of large objects, and the aesthetic, morphological and mechanical properties of these components were assessed. The findings of the present study will support the development of cheaper, lighter, recyclable PA6-based composites, suitable for the growing needs of the LFAM market.

## 2. Materials and Methods

### 2.1. Materials

The thermoplastic matrix used in this study was a commercial ECONYL ECO27, a chemically regenerated polyamide 6 supplied by Tessilquattro S.p.A. (Arco, TN, Italy) in pellet form (density = 1.13 g/cm^3^; melt volume rate at 250 °C and 2.16 kg = 28 cm^3^/10′ melting temperature = 221 °C). As reference, an ECONYL ECO 6G30 NAT was used, a commercial compound constituted by ECONYL ECO27 with the addition of 30 wt% of glass fibers. This product was provided by Tessilquattro S.p.A. in pellet form (density = 1.35 g/cm^3^; melt volume rate at 275 °C and 2.16 kg = 15 cm^3^/10′; melting temperature = 220 °C). As reported in the introduction, different kinds of natural fillers, supplied in powder form, were considered in the present study. Table 1 summarizes the key characteristics of these fillers, as reported in the suppliers’ datasheets.

### 2.2. Sample Preparation

Materials were dried in an oven at 60 °C for 48 h before being used. Neat PA6 and PA6 reinforced with 30% glass fibers were melt-compounded using a Thermo Haake Rheomix 600 internal mixer (ThermoFisher Scientific Inc., Watham, MA, USA) equipped with counter-rotating rotors, operating at 60 rpm at a temperature of 235 °C for 6 min. The different fillers were added to neat PA6 after 4 min, and the mixtures were then left to mix for 2 min. The processing parameters and the filler concentrations were selected after preliminary analysis, taking into account the processability of the materials and the necessity of avoiding thermal degradation. Specifically, the maximum filler content was limited to 10 wt% to guarantee the feasibility of the chemical recycling at the end of the material’s life cycle, according to information provided by the chemical recycling plant. In the case of silica, the content was further limited to 5 wt% due to its low density, which can lead to challenges in handling and feeding, including inconsistent dosing and difficulty entering the hopper during processing. The resulting blends were then granulated using a Piovan^®^ RN166/1 granulator (Piovan SpA, Venice, Italy) to obtain pellets of approximately 2 mm. The produced samples are listed in Table 2.

**Figure 1:**
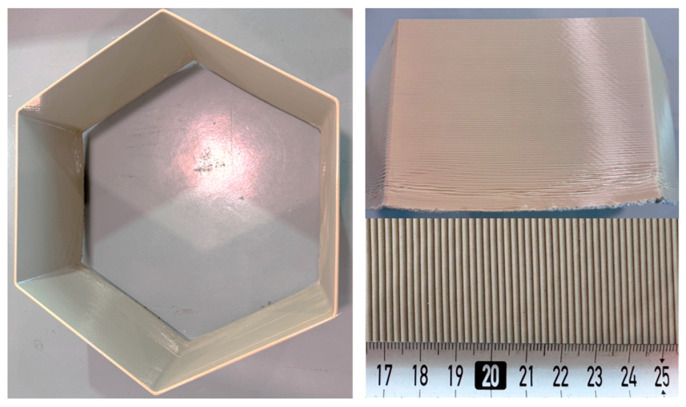
Representative images of a printed LFAM formulation (PA6-10-Clay).

The most suitable PA6-based compositions were then utilized in the form of pellets to print large components through LFAM. Specifically, different printing attempts were carried out using a HERON AM printer owned by Caracol AM (Barlassina, Italy). This system was constituted by a six-axis robot arm, KR 180 R2900 Prime (KUKA SE & Co. KGaA, Augsburg, Germany) and a High-Accuracy (HA) extruder, equipped with a 5 mm diameter nozzle. The printing parameters reported in Table 3 were optimized for each material to ensure stable extrusion, continuous bead deposition and acceptable print quality, taking into account differences in melt viscosity and flow behavior among the formulations. A representative picture of a printed LFAM component is shown in Figure 1. The printed samples were investigated from an aesthetic (i.e., defect inspection through light microscopy), morphological (i.e., FESEM analysis), and mechanical (i.e., flexural tests) point of view.

### 2.3. Experimental Techniques

#### 2.3.1. Rheological Characterization

The rheological properties of the samples were investigated using a Discovery hybrid rheometer DHR-2 (TA Instruments, New Castle, DE, USA) operating in parallel plate configuration, using a gap distance of 1 mm and a plate diameter of 20 mm. The tests were carried out at a constant temperature of 250 °C in oscillatory mode with a frequency range between 0.05 and 600 rad/s. From these tests, the storage modulus (*G′*), loss modulus (*G″*), loss factor (*tanδ*), and complex viscosity (η*) as a function of the angular frequency were evaluated. Moreover, the shear-thinning exponent (*n*) was calculated according to Equation (1) [34]:

(1) $$\eta^{*}=C{\dot{\gamma }}^{n-1}$$

where *C* is the consistency index and $\dot{\gamma }$ is the shear rate. Fluids with a value of *n* equal to 1 are identified as Newtonian fluids. Conversely, as the values of *n* decrease, the shear-thinning behavior of the fluid becomes more pronounced. These values were determined as the slope of the fitting curves of the log–log plot of complex viscosity as a function of angular frequency. The results of the dynamic rheological measurements were utilized to perform a preliminary selection of the most promising formulations.

The evaluation of the melt flow index (MFI) was performed using a Dynisco Melt Flow Indexer (Dynisco, Franklin, MA, USA) operating at a temperature of 235 °C, applying a load of 2.16 kg. A cut time of 5 s was used, and 10 specimens were measured for each formulation.

#### 2.3.2. Morphological Analysis of the Natural Fillers

The morphology of the selected fillers was investigated using a Zeiss Supra 40 field emission scanning electron microscope (FESEM) (Carl Zeiss AG, Oberkochen, Germany) operating at an accelerating voltage of 4 kV inside a chamber under a vacuum of 10^−6^ Torr. Prior to the observations, the materials were dried in a ventilated oven at 50 °C for 48 h and then coated with a thin electrically conductive platinum/palladium (Pt/Pd) layer. From the micrographs, the particle size was measured by using ImageJ^®^ software (version 1.53a).

#### 2.3.3. Thermal Properties

Thermogravimetric analysis (TGA) was carried out using a Mettler TA50 machine (Mettler Toledo Inc., Columbus, OH, USA). Specimens of approx. 15 mg were subjected to a heating ramp from 30 °C to 700 °C at 10 °C/min, under a constant air flow of 10 mL/min. This test allowed the determination of the temperature corresponding to a mass loss of 5% (*T*_5%_), the degradation temperature (*T_d_*) associated with the maximum of the first derivative of the TGA thermogram (DTG), and the residual mass at 700 °C (*m_R_*_,700_).

Differential scanning calorimetry (DSC) was performed using a Mettler DSC5+ calorimeter (Mettler Toledo Inc., Columbus, OH, USA). Specimens of approx. 10 mg were sealed in aluminum crucibles and subjected to a heating/cooling/heating cycle between 30 °C and 250 °C at 10 °C/min, under a constant nitrogen flow of 100 mL/min. The tests enabled the measurement of the glass transition temperature (*T_g_*), the melting temperature (*T_m_*) and the crystallization temperature (*T_c_*), as well as the corresponding enthalpies (Δ*H_m_* and Δ*H_c_*) of the samples. Furthermore, the degree of crystallinity of PA6 was calculated according to Equation (2):

(2) $$\chi =\frac{\Delta H_{m}\;-\;\Delta H_{cc}}{\Delta H_{m,th}\;\cdot \;\omega }\;\cdot \;100$$

where Δ*H_cc_* is the cold crystallization enthalpy of the PA6, Δ*H*_*m*,*th*_ is the enthalpy of melting of fully crystalline PA6, equal to 229.7 J/g [35], and *ω* is the weight fraction of PA6 in the composites.

Thermal conductivity (λ) was indirectly determined according to Equation (3):

(3) $$\lambda =\alpha \;\cdot \;c_{p}\;\cdot \;\rho$$

where *α* is the thermal diffusivity, c*_p_* is the specific heat and *ρ* is the bulk density of the analyzed specimens. The thermal diffusivity at 30 °C was determined by using a Neztsch Laser Flash Analyzer (LFA) 446 (Netzsch GmbH, Selb, Germany). A laser signal of 250 V was imposed using a pulse width of 0.6 ms. For these measurements, 1 mm thick discs with a diameter of 12.5 mm were produced by hot pressing the granules with a Carver^®^ Hot Press (Carver Inc. Wabash, IN, USA), at 250 °C for 3 min without load and 6 min with a load of 3 tons (corresponding to a pressure of 50 MPa). The discs were coated with a layer of graphite prior to measurement. Three specimens were tested for each composition, with five measurements taken on each specimen. The specific heat capacity at constant pressure (c_p_) was determined in the solid state with a sapphire reference according to the ASTM E1269-11 standard. One specimen of 8 mg for each sample was analyzed using a Mettler DSC5+ (Mettler-Toledo, Greifensee, Switzerland) calorimeter at a temperature of 30 °C by imposing a heating ramp between 25 and 35 °C, at 1 °C/min, under a constant nitrogen flow of 100 mL/min. The reference and sample crucibles were selected with a mass difference < 0.02 mg for all the tests; no lid was applied on the crucible, and a blank curve subtraction was performed. In order to guarantee good contact between the specimen and the lid, a heating scan up to 250 °C was performed prior to the measurement to melt the material. The density (*ρ*) was determined at 20 °C as the ratio between the mass and geometrical volume. Three specimens were tested for each composition.

#### 2.3.4. Characterization of the Components Produced Through LFAM

The evaluation of visual defects of the components produced through LFAM was carried out using a Nikon SMZ25 light microscope (Nikon, Tokyo, Japan), equipped with a Nikon DS-Fi2 digital camera (Nikon, Tokyo, Japan). Warping (*Wp*) was qualitatively evaluated in the correspondence of the corners of the printed component according to Equation (4):

(4) $$\mathrm{Wp}=\;\frac{H\;-\;Hc}{H}$$

where *H* and *Hc* are the average height and the height corresponding to the corners of the printed LFAM component (Figure 1), respectively.

Micrographs of cryo-fractured cross-sections of the printed samples were obtained using the Zeiss Supra 40 field emission scanning electron microscope, operating at an accelerating voltage of 3 kV inside a chamber under a vacuum of 10^−6^ Torr. Prior to the observations, the samples were dried in a fan oven at 50 °C for 48 h and then coated with a thin electrically conductive platinum/palladium (Pt/Pd) layer.

**Figure 2:**
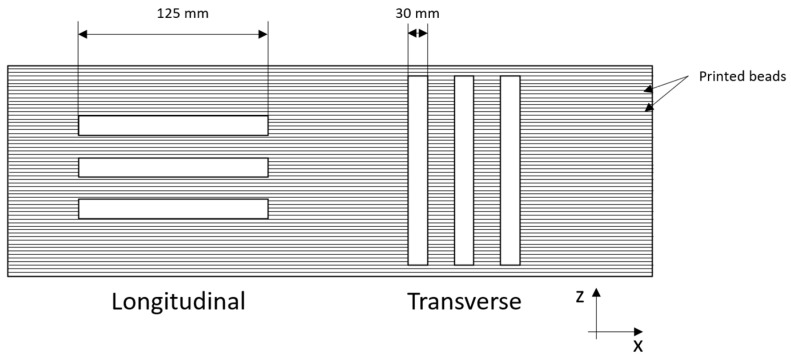
Schematic illustration of the specimens cut in longitudinal and transverse directions for quasi-static flexural tests.

Three-point bending tests were performed using an Instron 5969 machine (Instron^®^ Mechanical Testing Systems, Norwood, MA, USA) equipped with a load cell of 50 kN. A span-to-depth ratio of 16 was used. The tests were performed at constant temperature of 24 °C and at a speed of 5 mm/min. Specimens with dimensions of 125 × 30 × 7 mm^3^ were cut from the LFAM-printed component (Figure 1) in two different directions, i.e., the longitudinal direction (parallel to the motion of the extruder) and the transverse direction (perpendicular to the plane of the extruded layers), as shown in Figure 2. These tests were carried out to determine the flexural modulus (E_f_), the maximum flexural strength (σ_f,max_) and the flexural strain at maximum load (ε_f,max_) according to ASTM D790. For the samples that did not break during testing, the maximum flexural strength was defined as the value corresponding to the 5% strain. For each composition, at least five specimens were tested in both directions.

#### 2.3.5. Statistical Analysis

All the results are presented as mean ± standard error of the mean. Data were analyzed by using one-way analysis of variance (ANOVA) with a significance level of 0.05. Pairwise differences between treatments were assessed using the post hoc Tukey’s test.

## 3. Results and Discussion

### 3.1. Rheological Characterization

**Figure 3:**
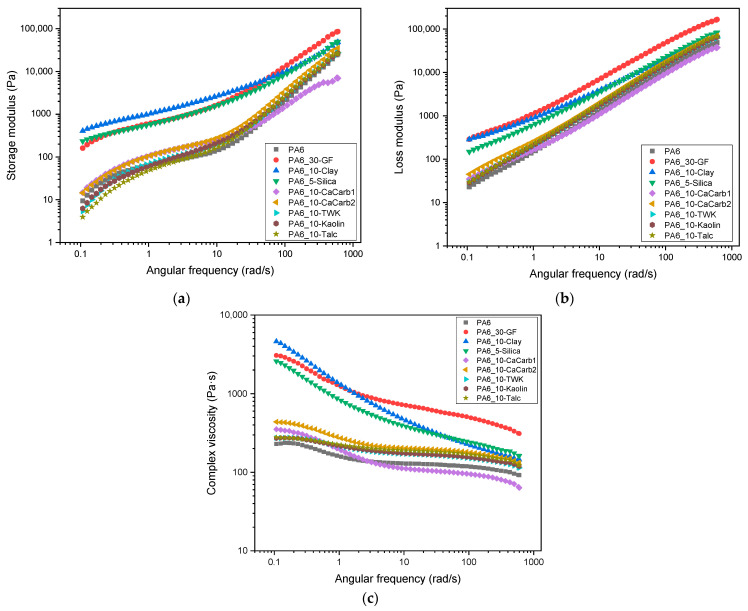
Dynamic rheological behavior of the produced PA6-based samples (T = 250 °C). Trends of (**a**) storage modulus, (**b**) loss modulus and (**c**) complex viscosity as a function of the angular frequency.

Figure 3a–c show the results of the dynamic rheological analyses of the different samples.

As reported in Figure 3a,b, it can be observed that the rheological behavior of all the samples is dominated by the viscous component, as the G″ values are higher than the respective G′ values over the entire frequency range. Furthermore, PA6_5-Silica and PA6_10-Clay samples exhibit similar viscoelastic properties to those of GF-reinforced samples. Conversely, the samples filled with talc, kaolin, wollastonite and calcium carbonate exhibit a rheological behavior more similar to neat PA6, indicating a less pronounced stiffening effect in the molten state. From Figure 3c it can be noticed that the presence of the additives leads to an increase in the complex viscosity, especially at lower frequencies, due to the occurrence of physical interactions between the polymer matrix and the fillers, which hinder the mobility of PA6 macromolecules and increase the resistance to flow. Indeed, most of the filled samples exhibit a more evident shear-thinning behavior, characterized by a stronger decrease in viscosity with increasing frequency. At higher frequencies, the PA6_30-GF sample exhibits a markedly higher viscosity compared to the natural filler-reinforced composites. Specifically, this behavior is primarily attributed to the high aspect ratio (length/diameter) of glass fibers which, combined with the significantly higher filler content (30 wt%) compared to that of the natural filler-based composites (≤10 wt%), increases the effective solid volume fraction of the melt. For a molten polymer to be successfully extruded through a nozzle for LFAM applications, the materials must exhibit a strong shear-thinning behavior [36]. The *n* values calculated according to Equation (1) range from 0.6 to 0.8 for PA6_30-GF, PA6_5-Silica, PA6_10-Clay, and PA6_10-CaCarb1, while all the other samples present values close to 0.9, very similar to that of neat PA6 (0.91). Given the need to narrow down the field of research and considering rheological behavior as the main choice parameter, only samples with lower *n* values (PA6_10-Clay, PA6_5-Silica, and PA6_10-CaCarb1) were selected for further investigation.

**Figure 4:**
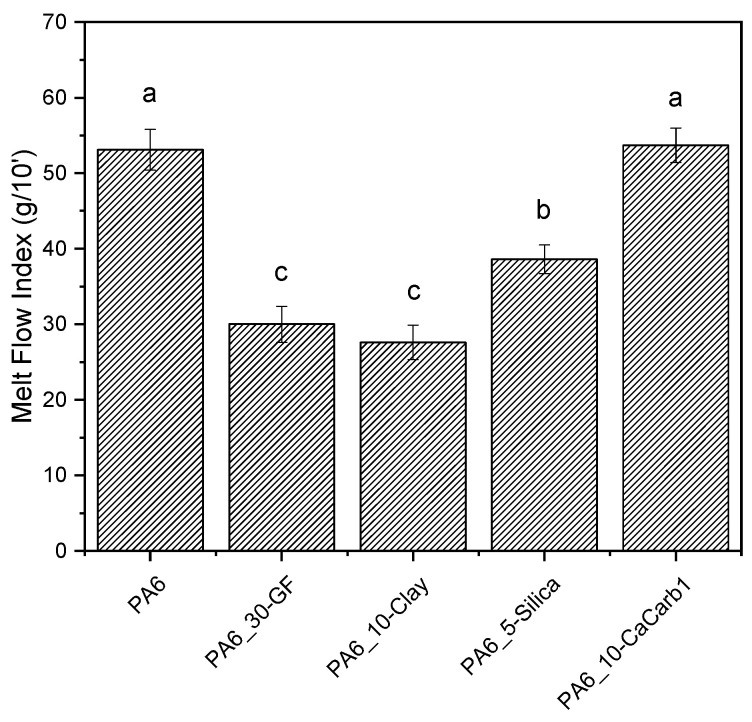
MFI results of neat PA6 and the relative composites (T = 235 °C, weight = 2.16 kg). Different letters indicate that results are statistically different (*p* < 0.05).

MFI tests were carried out to confirm the results of the dynamic rheological analysis on the selected formulations and to obtain data on the processing properties for use during production. The MFI results reported in Figure 4 highlight the strong influence of the filler type on the rheological behavior of the PA6 composites.

Specifically, it can be seen that the GF-reinforced sample exhibits low MFI values (−43% compared to neat PA6), indicating a substantial increase in the melt viscosity due to the high aspect ratio of fibers and high filler loading, as also observed from rheological analysis. Among the natural filler-based composites, PA6_10-Clay shows the lowest MFI values, corresponding to a reduction of 48% relative to neat PA6, with no statistically significant difference compared with the PA6_30-GF reference. The PA6_5-Silica sample shows an intermediate decrease in MFI of approximately 27%, while PA6_10-CaCCarb1 presents an MFI comparable to that of neat PA6, with no statistically significant difference, indicating a negligible effect of this filler on the melt viscosity.

### 3.2. Morphological Analysis of the Natural Fillers

**Figure 5:**
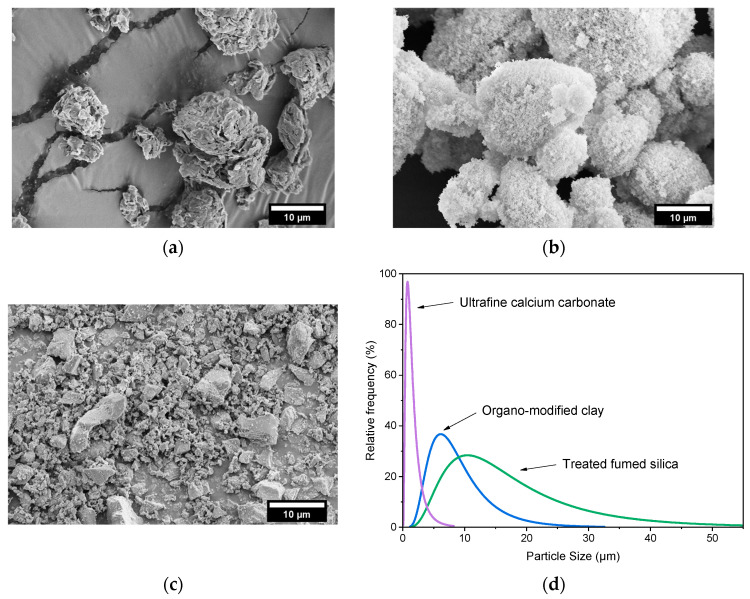
FESEM micrographs of the selected fillers: (**a**) trialkyl ammonium phyllosilicate BYK 4255; (**b**) hexadecylsilane-treated fumed silica Aerosil^®^ R816; (**c**) ultrafine calcium carbonate OM55AV; (**d**) filler size distribution.

Figure 5a–d show the FESEM micrographs of the selected natural fillers, used as reinforcement for PA6, together with their particle size distribution.

Figure 5a shows that the organo-modified clay has a morphology characterized by irregular agglomerates, with a mean particle size of 9.2 ± 6.1 µm. The treated fumed silica (Figure 5b) exhibits a more rounded particle morphology, although characterized by a broader size distribution and a higher mean particle size (18.5 ± 12.9 µm). Conversely, ultrafine calcium carbonate particles show an irregular shape, with a narrower size distribution and a smaller average particle size (1.7 ± 1.3 µm). These morphological observations are consistent with the rheological results. Indeed, the larger agglomerates of organo-modified clay and fumed silica can promote particle–particle interactions and the formation of filler networks within the polymer melt, thereby significantly affecting viscoelastic behavior at the molten state and the melt flow index values [37].

### 3.3. Thermal Properties

**Figure 6:**
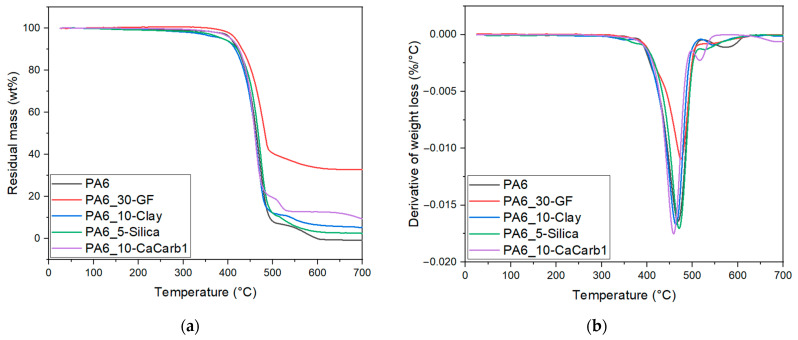
Results of TGA measurement on neat PA6 and the relative composites. Trends of (**a**) residual mass and (**b**) weight loss derivative as a function of the temperature.

Figure 6 and Table 4 report the results of TGA tests performed on the neat PA6 and the relative composite samples.

From the curves shown in Figure 6a,b, it can be seen that there is a substantial similarity in the thermal behavior of all samples, with the typical two-step decomposition behavior of PA6 in air [36,38,39]. Indeed, the first main degradation occurs between 400 and 500 °C, while the second small peak/shoulder is also visible between 500 and 600 °C due to the possible oxidation of residual char formed during the main decomposition step [39]. The GF-reinforced sample exhibits a slightly higher T_d1_ due to the high thermal stability of the glass fibers, which hinder the diffusion of degradation products [40]. For natural-filled samples, the temperature corresponding to a 5% mass loss and the maximum degradation rate temperatures are only marginally affected by the presence of the fillers, indicating that none of them significantly alters the thermal stability of the composites. As reported in Table 4, differences among the samples become evident primarily in the residual mass at 700 °C. As expected, the GF-reinforced sample exhibits the highest residual mass, reflecting its higher inorganic content, while the natural filler composites show values consistent with their nominal filler concentration. Overall, these results demonstrate that the incorporation of natural fillers does not substantially change the thermal stability of PA6, making them suitable for LFAM processing at relatively high temperatures (230–250 °C).

**Figure 7:**
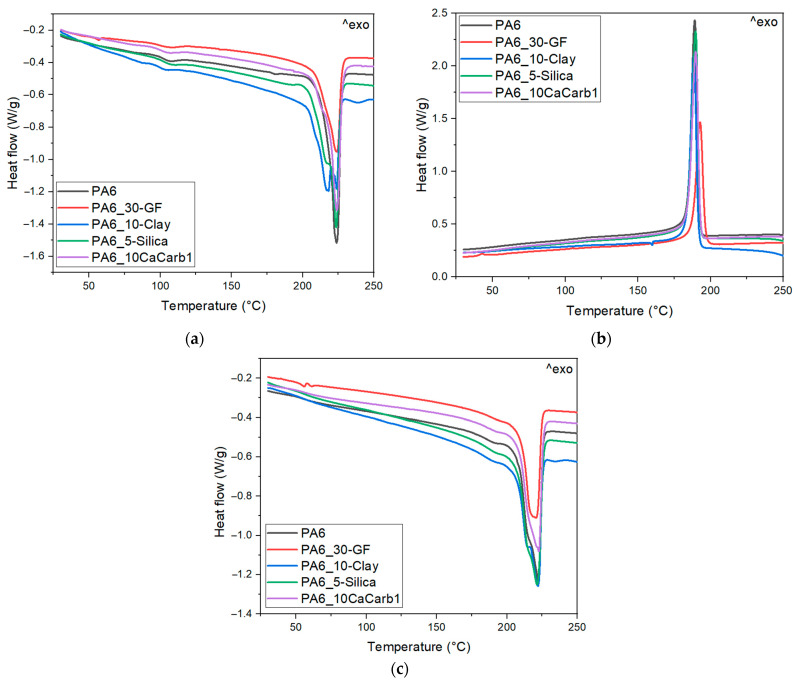
Results of DSC measurements on neat PA6 and the relative composites: (**a**) first heating, (**b**) cooling, and (**c**) second heating scans.

Figure 7a–c and Table 5 report the results of the DSC tests performed on neat PA6 and the relative composites.

The DSC curves shown in Figure 7a–c reveal a substantial similarity in the thermal behavior of all investigated samples. The melting and crystallization temperatures are substantially unaffected by the addition of fillers, while the values of melting and crystallization enthalpies are consistent with the concentration of the polymer matrix. The values of glass transition temperatures during the first heating scan are higher than those obtained during the second heating stage, due to the thermal history and physical aging of the processed materials. Indeed, T_g2_ values reflect the intrinsic glass transition of PA6 composites following the removal of processing-induced constraints. Overall, the T_g_ values remain essentially unchanged, although a slight increase is observed for the GF- and clay-filled composites, suggesting a modest reduction in polymer chain mobility due to filler–matrix interactions. Some differences among the samples are mainly observed in the degree of crystallinity. The neat PA6 sample presents X1 values of around 25% and X2 values of around 29%, while the addition of fillers generally leads to a slight increase in crystallinity. This behavior indicates a possible nucleating effect induced by the fillers, which can facilitate crystal formation during cooling.

For LFAM applications, a sufficiently large temperature interval between the melting temperature and the crystallization temperature is advantageous, as it extends the processing window in which the polymer remains in a molten state. This leads to a better interlayer diffusion and stronger welding between successive deposited layers before crystallization-induced solidification occurs [41]. In this case, the addition of natural fillers does not significantly affect the processing window of PA6, suggesting that the parameters typically used for PA6 in LFAM (e.g., deposition speed) can be maintained also for the developed composites.

Table 6 reports the results of the specific heat, thermal diffusivity, density and thermal conductivity of the produced samples.

From Table 6, it can be determined that the specific heat capacity of the filled composites is generally higher than that of neat PA6, reflecting the higher heat capacity of inorganic fillers compared to the PA6 matrix [42]. In the thermal diffusivity results, minimal variations are observed between the samples. Density measurements confirm the expected increase in density upon filler addition, with the GF-reinforced composite displaying the highest values due to its greater filler content. The combined effects of specific heat, thermal diffusivity and density are reflected in the thermal conductivity values. Overall, the addition of fillers leads to an increase in the thermal conductivity, in agreement with the results reported in the literature [43,44]. The highest values are observed for the GF- and calcium carbonate-reinforced samples (0.45 and 0.42 W/mK, respectively). For LFAM applications, the increased thermal conductivity suggests more efficient heat dissipation during printing, which can promote faster cooling and improved dimensional stability of the deposited beads, thereby reducing the risk of part distortion [11]. Conversely, the clay-reinforced sample exhibits a thermal conductivity comparable to that of neat PA6, suggesting that this filler does not significantly promote heat transfer within the material. This may result in slower cooling, which in turn requires careful control of printing parameters to prevent excessive heat accumulation.

### 3.4. Characterization of the Components Produced Through LFAM

#### 3.4.1. Evaluation of Visual Defects

**Figure 8:**
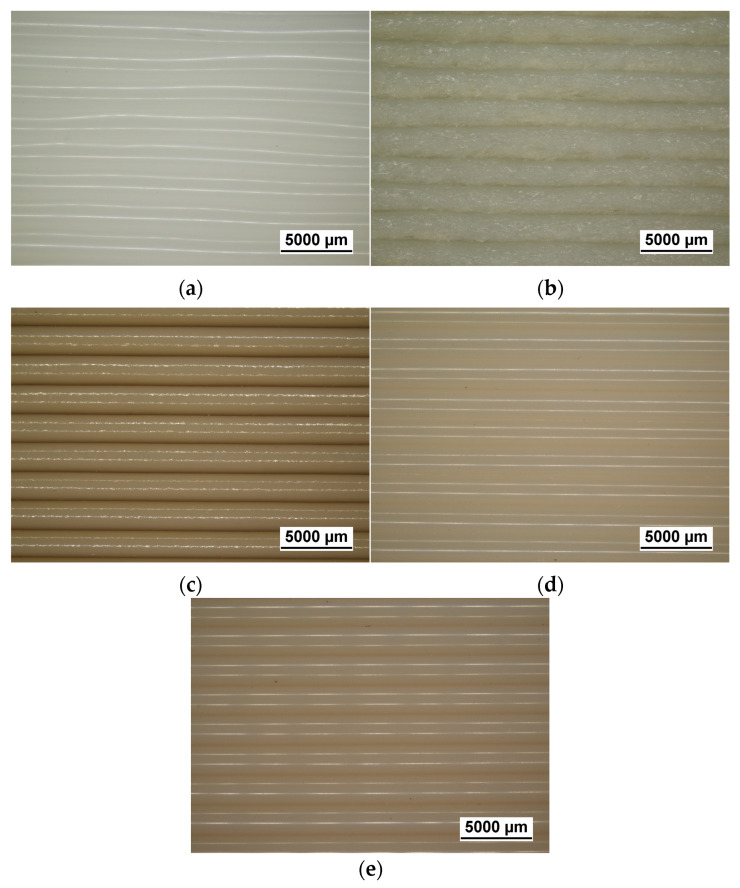
Light microscope images of the LFAM-printed samples: (**a**) PA6; (**b**) PA6-30GF; (**c**) PA6-10-Clay; (**d**) PA6-5-Silica; (**e**) PA6-10-CaCarb1.

Figure 8a–e show representative light microscope images of the samples printed through LFAM in order to assess their printing quality.

The light microscopy images in Figure 8a–e show generally good print quality for all compositions, with clearly distinguishable and well-aligned deposited beads along the printing direction. Specifically, the neat PA6 sample (Figure 8a) exhibits a relatively smooth surface, although some local curvature and irregularities can be observed. These features are likely related to the low melt strength and anisotropic shrinkage typical of semicrystalline polymers during cooling [4]. The GF-reinforced sample (Figure 8b) displays a noticeably rougher surface and irregular filament boundaries due to the high glass fiber content, which may have disrupted polymer melt continuity, consistent with the rheological results observed for this sample. The natural filler-reinforced composites (Figure 8c–e) show a well-defined filament morphology, with a relatively smooth surface and uniform bead stacking, indicating good filler dispersion. The average warping percentage, qualitatively assessed at the six corners of the printed LFAM components, is 3.1 ± 0.4% for the neat PA6 formulation, 1.5 ± 0.4% for PA6-30GF, 3.7 ± 0.8% for PA6-10-Clay, 4.0 ± 0.3% for PA6-5-Silica, and 3.2 ± 0.9% for PA6-10-CaCarb. Given the need to minimize warping in LFAM applications, future research will focus on the incorporation of an elastomeric phase in the formulation that may improve printability and reduce geometric defects.

#### 3.4.2. Morphological Analysis

**Figure 9:**
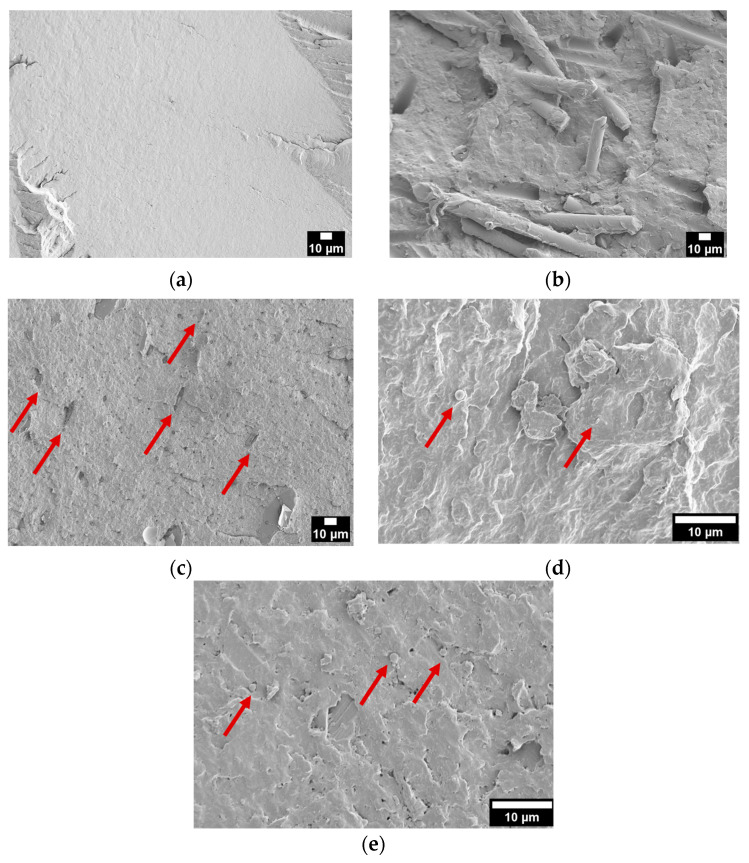
FESEM micrographs of the LFAM-printed samples (transverse direction): (**a**) PA6; (**b**) PA6-30GF; (**c**) PA6-10-Clay; (**d**) PA6-5-Silica; (**e**) PA6-10-CaCarb1. Red arrows indicate the presence of natural fillers.

Figure 9a–e show the FESEM micrographs of the cryo-fractured surfaces of the samples printed through LFAM and cut in the transverse direction.

Neat PA6 (Figure 9a) shows a relatively smooth and homogeneous fracture surface, characteristic of ductile polymers. In Figure 9b, it can be seen that the GF-reinforced composite exhibits exposed and partially pulled-out glass fibers, indicating that fracture propagation mainly occurred through matrix cracking and fiber–matrix interfacial debonding. The PA6-10-Clay sample (Figure 9c) displays a rather brittle fracture morphology with several micro-voids and localized defects. Indeed, the clay particles appear in the form of plate-like structures that show a preferential alignment along a common direction, likely corresponding to the printing direction. In the case of the PA6-5-Silica sample (Figure 9d), the observed morphology derives from the lower filler content compared to the other systems, which limits particle–particle interaction and agglomeration, favoring excellent filler dispersion. For calcium carbonate-reinforced composites (Figure 9e), the smaller average particle size (evaluated in Figure 5d) promotes a more uniform distribution within the matrix, reducing stress concentration sites and preventing the formation of large voids.

#### 3.4.3. Flexural Properties

**Figure 10:**
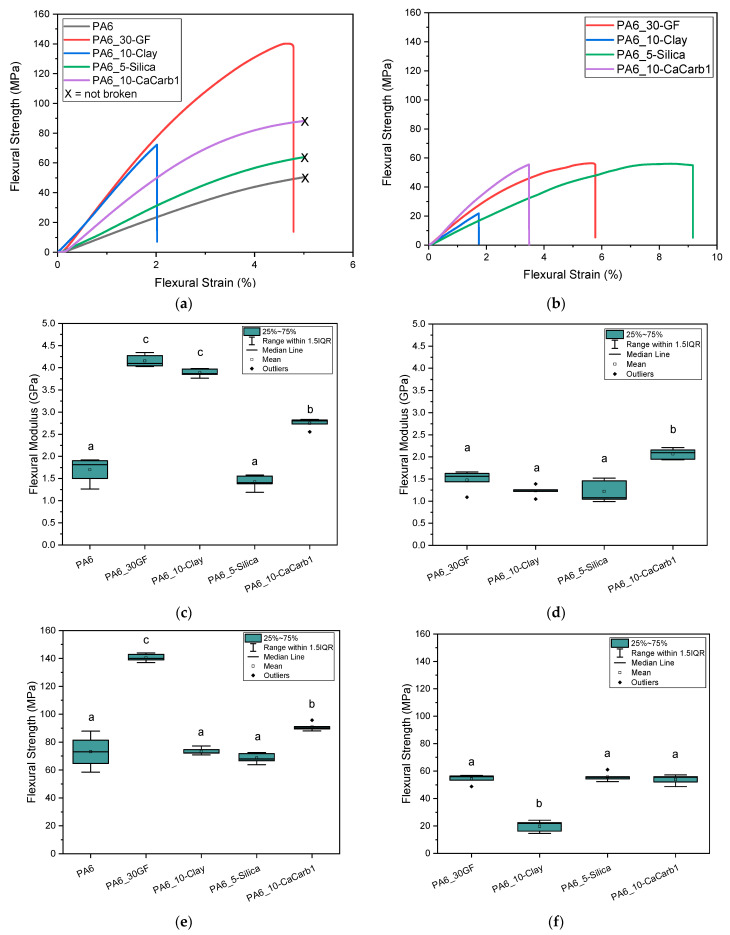
Results of flexural tests on LFAM-printed composites. (**a**) Representative flexural stress–strain curves in longitudinal printing direction; (**b**) representative flexural stress–strain curves in transverse printing direction; (**c**) flexural modulus in longitudinal printing direction; (**d**) flexural modulus in transverse printing direction; (**e**) flexural strength in longitudinal printing direction; (**f**) flexural strength in transverse printing direction. Different letters indicate that results are statistically different (*p* < 0.05).

Figure 10a–f summarize the flexural properties of printed specimens tested longitudinally and transversely to the printing direction. Table 7 reports the flexural properties of the samples normalized over their density to better highlight the possible advantages of using lightweight natural fillers. In the case of the transverse direction, the test could not be performed on the neat PA6 sample due to the presence of cracks parallel to the printing direction, which prevented the extraction of specimens in the transverse direction.

The flexural stress–strain curves obtained from specimens tested in the longitudinal direction of printing (Figure 10a) reveal that no fracture occurs for neat PA6 and for the silica- and calcium carbonate-reinforced samples. Instead, these materials exhibit a continuously increasing vertical deflection during measurement until the specimens started slipping on the supports, at which point the test was interrupted. As demonstrated in Figure 10c,e, in the parallel printing direction, the GF-reinforced sample exhibits a significantly higher flexural strength (+92% with respect to neat PA6) and modulus (+145% with respect to neat PA6), reflecting the combined effect of the high fiber loading and intrinsically high mechanical performance of glass fibers. Among the natural-filled composites, the PA6-10-CaCarb1 sample shows a significant increase (+30%) in flexural strength compared to the clay- and silica-filled composites, while maintaining a ductility comparable to that of neat PA6. The addition of clay to the PA6 matrix induces a strong embrittlement effect, as clay particles restrict polymer deformation and promote microcracking, even at low concentration [45]. This sample shows a flexural modulus comparable to that of GF-reinforced material thanks also to the strong filler orientation along the printing direction, observed from FESEM micrographs, while its ductility is significantly reduced. Moreover, the elastic modulus results in the longitudinal direction are comparable to those of 3D-printed short carbon fiber-reinforced PA6 composites [46]. For all the samples, the results obtained in the transverse direction (Figure 10b,d,f) show a marked reduction in the flexural strength and modulus, highlighting the intrinsic anisotropy of LFAM-printed parts [12,47]. This reduction is primarily associated with the weaker interlayer bonding and limited stress transfer across deposited beads [48]. In this orientation, the differences between the different fillers are less pronounced, with calcium carbonate- and silica-reinforced composites retaining a significantly higher flexural modulus and ductility, respectively. The strong reduction in mechanical performance for the PA6-10-Clay sample in this direction is again explained by the filler orientation, which contributes to enhancing the stiffness along the printing direction but, conversely, facilitates crack propagation along the platelet–matrix interface. Overall, these results demonstrate that, while the glass fiber reinforcement maximizes flexural strength and stiffness, primarily along the printing direction, selected natural fillers, especially organo-modified silica and calcium carbonate, can provide a more balanced mechanical response in both directions, thereby reducing anisotropy.

These outcomes are favorable, as they demonstrate the feasibility of substituting glass fibers with natural fillers in lower amounts, while maintaining mechanical properties suitable for a LFAM process and potentially ensuring the chemical recycling of the materials at the end of their service life.

## 4. Conclusions

This study investigated the influence of different natural fillers (i.e., clay, silica and calcium carbonate) as potential alternatives to glass fibers in PA6-based composites for Large-Format Additive Manufacturing applications in order to guarantee the chemical recyclability of the produced composites. Specifically, different PA6-based composites, with a natural filler content of ≤10 wt%, were developed and compared with 30 wt% GF-reinforced PA6 composites from a rheological and thermo-mechanical point of view. GF-reinforced samples showed the highest viscosity and lowest melt flow index due to the combined effect of the high fiber aspect ratio and elevated filler loading. Clay and silica also displayed an increase in melt viscosity as a result of their agglomerated morphology and higher particle size, whereas calcium carbonate showed a limited effect on rheological properties due to the smaller particle dimension. TGA and DSC analysis revealed that the thermal decomposition and melting behavior of the samples were not significantly affected by the filler addition, with variations observed only in the residual mass at 700 °C (due to filler content) and in the slight increase in crystallinity due to the nucleating effect provided by the filler introduction. Thermal conductivity measurements revealed that calcium carbonate-filled composites achieved λ values comparable to the GF-filled reference (≥0.42 W/mK), suggesting more efficient heat dissipation, which could promote faster cooling and a reduced risk of part distortion during printing. The selected compositions were then successfully used for the preliminarily printing of LFAM components that were evaluated from a morphological and mechanical point of view. Microscopy analysis confirmed a better printing quality for the natural-filled composites compared to the GF-reinforced sample. FESEM observations, carried out on the cryo-fractured surfaces of printed specimens, indicated a preferential platelet orientation for clay-filled samples along the printing direction. In contrast, silica- and calcium carbonate-reinforced composites showed a more homogeneous particle dispersion and limited void formation, attributed respectively to the lower filler content and smaller particle size. Flexural tests confirmed a strong anisotropy induced by the LFAM process, particularly in the GF- and clay-reinforced samples, whereas calcium carbonate and silica provided a more balanced mechanical response. These results are especially promising considering that the natural fillers were used at significantly lower loadings (≤10 wt%) compared to the 30 wt% glass fiber reference, yet they were still able to provide competitive mechanical performance, ensuring the printability of the components and the potential chemical recyclability at their end of life. This highlights the potential of properly selected natural fillers as viable and lightweight alternatives to conventional glass fiber reinforcement in LFAM applications. Future research will focus on optimizing the processing parameters in order to further improve the printing quality of large components. Moreover, the incorporation of a small amount of the elastomeric phase will be explored to further improve printability, enhance interlayer adhesion and reduce defect formation.

## Figures and Tables

**Table 1:** List of natural fillers considered in the present study, with their main characteristics.

Filler Name	Filler Type	Supplier	Physical Properties
BYK 4255	Trialkyl ammonium phyllosilicate	BYK-Chemie GmbH (Wesel, Germany)	Density: 1.88 g/mL Particle size: <40 µm
Aerosil^®^ R816	Hexadecylsilane treated fumed silica	Evonik Operations GmbH (Essen, Germany)	Tamped density: 60 g/L Surface area: 170–210 m^2^/g
OMYA OM55AV	Ultrafine calcium carbonate	Omya International A.G. (Oftringen, Swiss)	Specific gravity: 2.70 Particle < 2 µm: 56%
OMYA OM1AV	Calcium carbonate	Omya International A.G. (Oftringen, Swiss)	Specific gravity: 2.70 Particle < 2 µm: 21%
Crys-Talc^®^ 7 C	Ultrafine talc	Imerys S.A. (Paris, France)	Specific gravity: 2.78 Surface area: 16.9 m^2^/g
Talc T10	Ultrafine talc	Imerys S.A. (Paris, France)	Specific gravity: 2.70 Surface area: 15.0 m^2^/g
Nyglos^®^ M4W	Ultrafine wollastonite	Imerys S.A. (Paris, France)	Specific gravity: 2.90 Surface area: 2.7 m^2^/g
Polarite^™^ 102A	Aminosilane coated calcined kaolin	Imerys S.A. (Paris, France)	Specific gravity: 2.60 Surface area: 8.5 m^2^/g
Translink^®^ 445	Dehydroxylated calcined kaolin	BASF Corporation (Florham Park, NJ, USA)	Specific gravity: 2.63 Particle size: 1.4 µm

**Table 2:** List of prepared samples.

Sample	Filler Type	Filler Amount (wt%)
PA6	-	-
PA6_30-GF	Glass fibers	30
PA6_10-Clay	Trialkyl ammonium phyllosilicate	10
PA6_5-Silica	Hexadecylsilane treated fumed silica	5
PA6_10-CaCarb1	Ultrafine calcium carbonate	10
PA6_10-CaCarb2	Calcium carbonate	10
PA6_10-TWK	Ultrafine talc	1
	Ultrafine wollastonite	4.5
	Aminosilane coated calcined kaolin	4.5
PA6_10-Kaolin	Dehydroxylated calcined kaolin	10
PA6_10-Talc	Ultrafine talc	10

**Table 3:** Printing parameters for the selected samples.

Parameters	Deposition Speed (mm/s)	Layer Time (s)	Layer Height (mm)	Layer Width (mm)	Nozzle Temperature (°C)
PA6	66	36	2.0	6.7	230
PA6_30-GF	66	36	2.0	8.0	245
PA6-10-Clay	66	36	2.0	6.7	250
PA6-5-Silica	66	36	2.0	7.1	250
PA6-10-CaCarb1	66	36	2.0	6.5	235

**Table 4:** Results of the TGA measurements on neat PA6 and the relative composites.

Sample	T_5%_ (°C)	T_d1_ (°C)	T_d2_ (°C)	m_r_ (%)
PA6	404.3	468.3	575.2	0.0
PA6_30-GF	416.8	476.7	542.2	29.9
PA6_10-Clay	399.2	466.8	547.8	8.4
PA6_5-Silica	399.3	470.8	528.5	4.4
PA6_10-CaCarb1	407.0	462.7	516.8	9.4

T_5%_ = temperature at 5% of degradation; T_d1_, T_d2_ = temperatures of maximum degradation rate; m_r_ = residual mass at 700 °C.

**Table 5:** Results of the DSC measurements on neat PA6 and the relative composites.

Sample	T_g1_ (°C)	T_m1_ (°C)	∆H_m1_ (J/g)	χ_1_ (%)	T_c_ (°C)	∆H_c_ (J/g)	T_g2_ (°C)	T_m2_ (°C)	∆H_m2_ (J/g)	χ_2_ (%)
PA6	102.1	223.8	58.3	24.9	189.0	70.8	54.8	222.0	67.3	29.3
PA6_30-GF	99.3	223.7	49.5	30.8	192.7	49.5	59.2	220.7	52.3	32.5
PA6_10-Clay	100.5	220.2	57.8	28.0	188.5	65.7	58.2	222.2	65.5	31.7
PA6_5-Silica	102.5	223.6	57.9	26.1	189.6	68.9	55.8	221.4	68.3	31.3
PA6_10-CaCarb1	100.5	223.9	57.7	27.9	189.7	67.7	55.3	222.5	65.6	31.7

T_g1_, T_g2_ = glass transition temperature (first and second heating scan); T_m1_, ∆H_m1_ = melting temperature and enthalpy (first heating scan); T_c_, ∆H_c_ = crystallization temperature and enthalpy (cooling scan); T_m2_, ∆H_m2_ = melting temperature and enthalpy (second heating scan); χ_1_, χ_2_ = degree of crystallinity (first and second heating scan).

**Table 6:** Results of specific heat, thermal diffusivity, density and thermal conductivity of the produced samples.

Sample	c_p_ (J/g∙K)	α (mm^2^/s)	ρ (g/cm^3^)	λ (W/m∙K)
PA6	1.47 ± 0.01	0.21 ± 0.01	1.06 ± 0.03	0.33 ± 0.02
PA6_30-GF	1.50 ± 0.01	0.23 ± 0.01	1.31 ± 0.04	0.45 ± 0.02
PA6_10-Clay	1.56 ± 0.01	0.19 ± 0.01	1.17 ± 0.01	0.35 ± 0.02
PA6_5-Silica	1.65 ± 0.01	0.20 ± 0.01	1.14 ± 0.01	0.38 ± 0.01
PA6_10-CaCarb1	1.64 ± 0.01	0.22 ± 0.01	1.18 ± 0.02	0.42 ± 0.03

**Table 7:** Flexural properties of samples normalized over their density (ρ).

Sample	E/ρ (GPa/(g/cm^3^))		σ_f_/ρ (MPa/(g/cm^3^))	
	Longitudinal	Trasverse	Longitudinal	Trasverse
PA6	1.6 ± 0.3 ^b^	-	68.9 ± 11.3 ^a^	-
PA6_30-GF	3.2 ± 0.1 ^d^	1.1 ± 0.2 ^a^	107.3 ± 2.2 ^b^	41.5 ± 2.6 ^b^
PA6_10-Clay	3.3 ± 0.1 ^d^	1.1 ± 0.1 ^a^	62.7 ± 2.1 ^a^	16.9 ± 3.6 ^a^
PA6_5-Silica	1.2 ± 0.1 ^a^	1.1 ± 0.2 ^a^	60.1 ± 3.1 ^a^	48.9 ± 2.9 ^c^
PA6_10-CaCarb1	2.3 ± 0.1 ^c^	1.8 ± 0.1 ^b^	77.0 ± 2.4 ^a^	45.7 ± 2.9 ^bc^

Different letters indicate that results are statistically different (*p* < 0.05).

## Data Availability

Data are available on reasonable request by the corresponding author.

## Abbreviations

The following abbreviations are used in this manuscript:

PA6	Polyamide 6
GF	Glass fibers
LFAM	Large-Format Additive Manufacturing
FESEM	Field emission scanning electron microscopy
TGA	Thermogravimetric analysis
DSC	Differential scanning calorimetry
